# Autoinflammatory gene polymorphisms and susceptibility to UK juvenile idiopathic arthritis

**DOI:** 10.1186/1546-0096-11-14

**Published:** 2013-04-02

**Authors:** Anne Hinks, Paul Martin, Susan D Thompson, Marc Sudman, Carmel J Stock, Wendy Thomson, Thomas G Day, Jon Packham, Athimalaipet V Ramanan, Rachelle P Donn

**Affiliations:** 1 Arthritis Research UK Epidemiology Unit, Stopford Building, The University of Manchester, Manchester Academic Health Science Centre, School of Translational Medicine, Oxford Road, Manchester M13 9PT, UK; 2 Division of Rheumatology, Cincinnati Children’s Hospital Medical Center, 3333 Burnet Ave, Cincinnati, OH 45229, USA; 3 Centre for Paediatric and Adolescent Rheumatology, Windeyer Institute for Medical Sciences, University College London, London, UK; 4 Haywood Hospital, University Hospital of North Staffordshire, Stoke on Trent, Staffordshire ST4 7LN, UK; 5 Department of Paediatric Rheumatology, Bristol Royal Hospital for Children & Royal National Hospital for Rheumatic Diseases, Bath, UK

**Keywords:** Autoinflammatory genes, Juvenile idiopathic arthritis, *MVK*, *TNFRSF1A*, *IL1A*

## Abstract

**Background:**

To investigate the autoinflammatory hereditary periodic fever syndrome genes *MVK* and *TNFRSF1A,* and the *NLRP1* and *IL1* genes, for association with juvenile idiopathic arthritis (JIA).

**Methods:**

For *MVK, TNFRSF1A* and *NLRP1* pair-wise tagging SNPs across each gene were selected and for *IL1A* SNPs from a prior meta-analysis were included. 1054 UK Caucasian JIA patients were genotyped by Sequenom iPlex MassARRAY and allele and genotype frequencies compared with 5380 unrelated healthy UK Caucasian controls.

**Results:**

Four SNPs were significantly associated with UK JIA: rs2071374 within intron 4 of *IL1A* (ptrend=0.006), rs2228576 3’ of *TNFRSF1A* (ptrend=0.009) and 2 SNPs, rs11836136 and rs7957619, within *MVK* (ptrend=0.006, ptrend=0.005 respectively). In all cases the association appeared to be driven by the systemic-onset JIA (SoJIA) subtype. Genotype data for the two *MVK* SNPs was available in a validation cohort of 814 JIA (oligoarticular and RF negative polyarticular) cases and 3058 controls from the US. Replication was not confirmed, however, further suggesting that this association is specific to SoJIA.

**Conclusions:**

These findings extend the observations of the relevance of studying monogenic loci as candidates for complex diseases. We provide novel evidence of association of *MVK* and *TNFRSF1A* with UK JIA, specifically driven by association with SoJIA and further confirm that the *IL1A* SNP association with SoJIA is subtype specific. Replication is required in independent cohorts.

## Background

Juvenile idiopathic arthritis (JIA) is the most common chronic rheumatic disease of childhood. It occurs, by definition, before the age of 16 years, with a duration of more than six weeks. JIA is a heterogeneous condition, and has been divided into several subtypes based on clinical features, family history, and laboratory markers [1], though they all share chronic inflammation of one or more synovial joints.

The contribution of common variants of genes, in which mutations cause distinct, rare syndromes, to complex oligogenic phenotypes is increasingly recognised [2-6].

We have previously described associations between the psoriatic subtype of JIA and single-nucleotide polymorphisms (SNPs) in four genes responsible for monogenic hereditary periodic fever syndromes (HPFs) or autoinflammatory syndromes [7]. The HPF syndromes are a heterogeneous group of autoinflammatory diseases, unified by the common feature of episodes of unprovoked inflammation, along with other signs of systemic infection such as fever. Arthralgia or arthritis is often part of the presenting form [8]. They are caused by aberrant activation of the innate immune system, a mechanism that may also be relevant to JIA.

In the present study we have investigated the genes implicated in two additional HPF syndromes. These are *MVK*, located on chromosome 12q24, responsible for hyper-IgD syndrome (HIDS), and *TNFRSF1A*, located on chromosome 12p13.2, responsible for TNF receptor-associated periodic syndrome (TRAPS). HIDS, like all HPF syndromes, is characterized by periodic inflammation, but has high serum IgD level and an array of inflammatory symptoms including lymphadenopathy, rash, gastrointestinal upset and arthritis [9]. Mutations in *MVK* are also responsible for the severe monogenic metabolic disorder mevalonic aciduria. TRAPS is the most common autosomal dominant HPF syndrome and is distinguished clinically by particularly long duration of attacks. Arthralgia and non-erosive synovitis are among the most common manifestations [10].

In addition, we have studied *NLRP1,* which encodes NACHT leucine-rich-repeat protein 1, a regulator of the innate immune system. NLRP1 provides a scaffold for the assembly of the inflammasome that activates caspases 1 and 5, which subsequently promote the processing and maturation of the inflammatory cytokines pro-IL-1β, IL-18 and IL-33 [11]. *NLRP1* is a homologue of *NLRP3*, one of the genes found previously to be associated with psoriatic JIA [7]. SNPs in *NLRP1* have recently been associated with vitiligo-associated autoimmune disease, autoimmune Addison’s disease, and type 1 diabetes [12,13].

Interleukin 1 (IL-1) helps coordinate the immune system’s early response to exogenous and endogenous danger, serving as a prototypic alarm cytokine. Increased production of IL-1 is thought to be a fundamental component in the pathogenesis of the majority of the HPF syndromes [14]. Furthermore, the systemic-onset subtype of JIA (SoJIA) has been classified, in recent years, as an autoinflammatory disease. Leukocyte gene expression profiling of SoJIA patients identified a unique IL-1β signature when compared to controls, and which changed significantly following IL-1β blockade [15]. However, subsequent studies have failed to replicate the IL-1β signature [16,17]. Stock *et al.* described association between SoJIA and SNPs in the *IL-1 ligand* and *IL-1 receptor* clusters [18]. The relevance of these SNPs to other JIA subtypes has not previously been investigated.

We have utilised a large collection of UK Caucasian JIA samples and controls to determine SNP associations with the autoinflammatory disease related loci *MVK*, *TNFRSF1A, NLRP1, IL1 ligand* and *IL1 receptor.* Data collected from a US genome wide association study of JIA was available for validation of any significant findings.

## Methods

### Initial UK cohort - subjects

DNA was available for 1054 UK Caucasian JIA patients (332 males: 715 females) from three sources. The British Society for Paediatric and Adolescent Rheumatology (BSPAR) National Repository of JIA (n=654), a cohort of UK Caucasian patients with long-standing JIA (n=201), described previously [19] and a third cohort collected as part of the Childhood Arthritis prospective Study (CAPS), a prospective inception cohort study of JIA cases from 5 centres across UK (n=199) [20]. JIA cases were classified according to ILAR criteria [1]. The numbers per subtype were: systemic onset (n=165), persistent oligoarthritis (n=276), extended oligoarthritis (n=143), rheumatoid factor (RF) negative polyarthritis (n=208), RF positive polyarthritis (n=67), enthesitis related JIA (n=64), psoriatic JIA (n=74) and unclassified (n=57). Additional control DNA samples for genotyping (n=794) were recruited from blood donors, or from the 1958 Birth Cohort (n=1717).

The SoJIA patients included in this present study are the same as those previously reported on by Stock *et al.*[18].

All individuals were recruited with ethical approval and provided informed consent [North-West Multi-Centre Research Ethics Committee (MREC 99/8/84) and the University of Manchester Committee on the Ethics of Research on Human Beings].

### SNP selection

Five loci were selected for analysis (*MVK, TNFRSF1A, NLRP1, IL1 ligand and IL1 receptor clusters).*

For *MVK, TNFRSF1A* and *NLRP1* pair-wise tagging SNPs across each gene and within 10 kb 5’ and 3’ of it were selected using HapMap release 22 (http://www.hapmap.ncbi.nlm.gov) and the tagger function in Haploview version 4.1 (http://www.broadinstitute.org/haploview/haploview), using an r^2^ cutoff ≥ 0.8 and MAF ≥ 0.05.

For the *IL1 receptor* and *IL1 ligand* clusters the four SNPs that were previously associated in a two-stage meta-analysis with SoJIA were selected for genotyping [18]. In the *IL1 receptor* cluster, rs2190360, which has r^2^=0.96 with rs12712122 was selected. In the *IL1 ligand* cluster, the three SNPs were rs6712572, rs2071374 and rs1688075.

### Genotyping

SNP genotyping was performed using the Sequenom iPlex® MassARRAY platform according to manufacturers instructions (Sequenom, San Diego, CA. http://www.sequenom.com/). A 90% sample quality control (QC) rate and 90% SNP genotyping success rate was imposed on the analysis.

### Control genotyping data

All SNPs, except rs6712572, rs1688075 and rs1295288, were genotyped as part of Wellcome Trust case control consortium 2 (WTCCC2). Some SNPs have been genotyped using the Affymetrix platform, others the Illumina platform and some have been genotyped on both, where this is the case the Affymetrix control data has been used preferentially, because of the larger cohort. Sample size for the WTCCC2 Affymetrix control data =5380, for the WTCCC2 Illumina control data =5200. The SNPs rs6712572 and rs1688075 were genotyped in controls from blood donors and general practitioner records (n=794) and rs1295288 was genotyped in 1958 Birth Cohort controls (n=1717).

### Validation cohort

For validation of the findings in the UK cohort genotype information was available from a replication cohort of 814 JIA cases and 3058 controls from the US. For the JIA children, with polyarticular (RF negative) and oligoarticular JIA, approximately 95% of the cases were recruited at the Cincinnati Children's Hospital Medical Center (CCHMC) or as part of a NIAMS supported JIA affected sibpair registry. The remaining cases were contributed by collaborating centers which included Children's Hospital of Wisconsin, Schneider Children's Hospital and Children's Hospital of Philadelphia. The numbers per subtype were: persistent oligoarthritis (n=343), extended oligoarthritis (n=118) and rheumatoid factor (RF) negative polyarthritis (n=353). The control cohort (n=3058) comprised 658 healthy children aged between 3–18 years, recruited from the general population to represent the geographical region served by CCHMC. In addition 2400 controls of European ancestry were genotyped at the Broad Institute on the Affymetrix SNP Array 6.0 as part of the Molecular Genetics of Schizophrenia (MGS) genome wide association study. Both cases and controls were run through principal component analysis to look for outliers, and any outliers excluded from analysis. SNPs were genotyped using the Affymetrix Genome-Wide Human SNP Array 6.0. The study had full ethical committee approval (CCHMC IRB) and was fully compliant with the Declaration of Helsinki.

### Statistical analysis

Power calculations were performed using QUANTO [21], calculations assumed a log-additive model and alpha value of 0.05. Genotype and allele frequencies were compared between cases with JIA and controls using the Cochrane-Armitage trend test implemented in PLINK [22] and allelic odds ratios (ORs) and their 95% confidence intervals (CIs) calculated. JIA is a phenotypically heterogeneous disease and can be classified into more clinically homogeneous diseases using the ILAR classification criteria [1]. Therefore SNPs were also analysed stratified by ILAR subtype, whereby each JIA subtype was individually compared to controls. Conditional logistic regression analysis was performed using PLINK.

## Results

After QC the dataset for analysis from the initial UK cohort comprised 1017 JIA cases and 5380 controls. Of the 41 SNPs genotyped, 2 SNPs failed SNP QC in the cases and 2 failed WTCCC2 control QC, these SNPs were dropped, leaving 37 SNPs for analysis. There was an average coverage of all genes, after QC, of >86%.

This study had >80% to detect an effect >1.25 for SNPs with allele frequencies >0.1.

Results for all SNPs studied in the total JIA group compared with controls are presented in Additional file 1: Table S1. The results for individual JIA subgroups compared with controls are presented in Additional file 1: Tables S2-S4.

**Figure 1:**
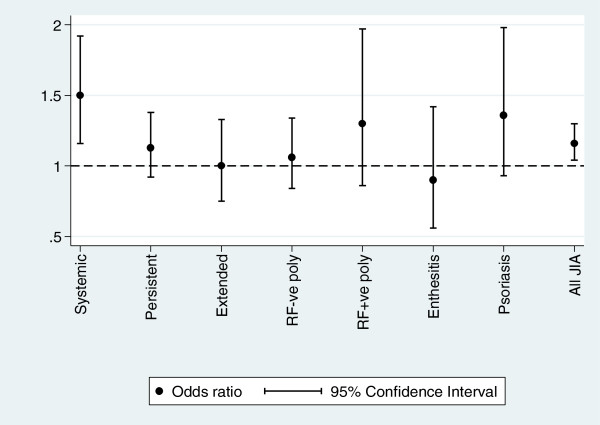
**Odds ratio and 95% CI plots for the *IL1A *SNP rs2071374 in UK JIA.** Allele frequencies for each JIA subtype was compared with that of the controls and the OR and 95% CI plotted. The comparison versus the total UK JIA cohort (All JIA) is also shown.

Of the 37 SNPs analysed four show significant association with JIA (Table 1). One SNP, rs2071374 in the *IL1 ligand* cluster showed significant association with JIA (ptrend=0.006 OR 1.16 95% CI 1.04-1.30) (Table 1). The association was with SoJIA (p=0.001 OR 1.5 95% CI 1.16-1.92). In addition, there was also a trend towards association in the psoriatic subtype (p=0.11 OR 1.36 95% CI 0.93-1.98). Odds ratio plot across all seven ILAR subtypes is shown in Figure 1. Overall, three of the four IL1-related SNPs tested in this study showed significant association with SoJIA (Additional file 1: Table S2), and when the dataset was reanalysed after removing the systemic subtype none of the SNPs remained significantly associated, further confirming the association as being largely driven by the SoJIA subtype (Additional file 1: Table S5). These SoJIA cases have been previously studied for the IL1-related SNPs by Stock *et al.*[18]. Our data shows no evidence for significant association of the IL1 ligand and IL1 receptor gene cluster with non SoJIA.

**Table 1:** SNPs significantly associated with JIA (p<0.05)

**SNP**	**CHR**	**Position**	**Gene**	**Major allele (allele 1)**	**Minor allele (allele 2)**	**MAF Case**	**MAF Control**	**pHWE Control**	**Case genotype frequencies**			**Control genotype frequencies**			**p-value**	**Odds ratio 95% CI**
									**1/1**	**1/2**	**2/2**	**1/1**	**1/2**	**2/2**		
									**(%)**	**(%)**	**(%)**	**(%)**	**(%)**	**(%)**		
rs2071374	2	113537352	*IL1A*	A	C	0.30	0.27	0.67	453	392	85	2751	2067	376	0.006	1.16 1.04-1.30
									(48.7)	(42.2)	(9.1)	(53.0)	(39.8)	(7.2)		
rs2228576	12	6457062	*TNFRSF1A*	G	A	0.33	0.36	0.43	414	419	94	2136	2349	678	0.009	0.87 0.78-0.97
									(44.7)	(45.2)	(10.1)	(41.4)	(45.5)	(13.1)		
rs11836136	12	110006512	*MVK*	A	G	0.29	0.26	1.00	460	380	74	2857	1992	347	0.006	1.17 1.04-1.30
									(50.3)	(41.6)	(8.1)	(55.0)	(38.3)	(6.7)		
rs7957619	12	110013879	*MVK*	G	A	0.14	0.11	0.33	699	210	21	4099	1027	73	0.005	1.231.07-1.43
									(75.2)	(22.6)	(2.3)	(78.8)	(19.8)	(1.4)		

CHR=chromosome, MAF=minor allele frequency, CI=confidence interval, pHWE= p-value for departure from Hardy-Weinburg equilibrium.

One SNP, rs2228576, at the 3’ end of the *TNFRSF1A,* but which actually lies within the adjacent gene sodium channel, non-voltage-gated 1 alpha (*SCNN1A*), was associated with JIA (ptrend=0.009 OR 0.87 95% CI 0.78-0.97) (Table 1).

**Figure 2:**
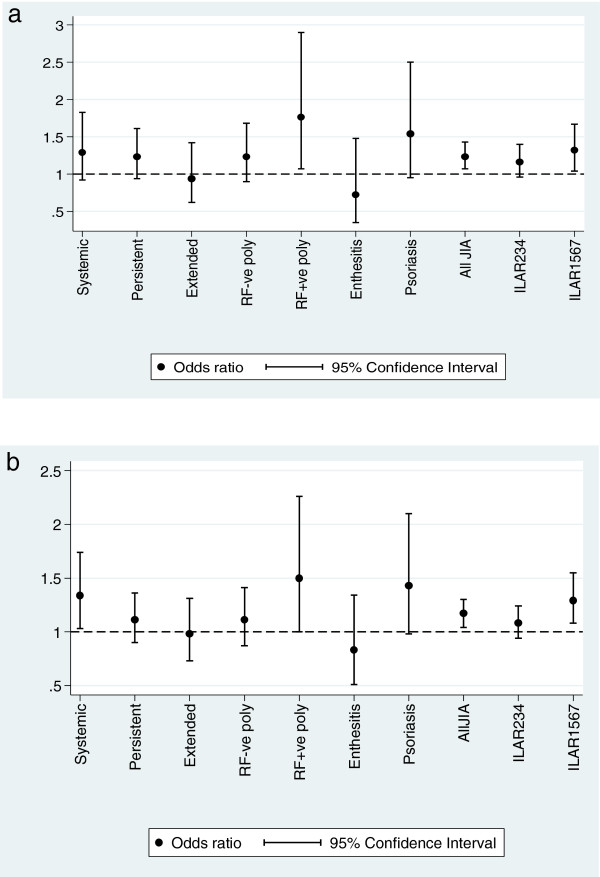
**Odds ratio and 95% CI plots for *MVK *SNPs in UK JIA.** Allele frequencies for each JIA subtype separately was compared with that of the controls and the OR and 95% CI plotted. In addition, the comparison versus the total UK JIA cohort (All JIA) is also shown. For both rs7957619 (Figure 2a) and for rs11836136 (Figure 2b) ILAR 234 comprising of persistent oligoarticular, extended oligoarticular and RF-ve polyarticular JIA, which represents those JIA subtypes included in the USA validation cohort, are not significantly associated with UK JIA. ILAR 1567 comprises the systemic onset, RF+ve polyarticular, enthesitis and psoriatic subtypes respectively.

Two SNPs in the *MVK* gene, rs1183616 (ptrend=0.006. OR 1.17, 95% CI 1.04-1.30) and rs7957619 (ptrend=0.005 OR 1.23 95% CI 1.07-1.43) were associated with JIA (Table 1). These two SNPs are in modest linkage disequilibrium (r^2^=0.36, D’=1). Logistic regression of the two SNPs, after conditioning on the most significant SNP, found that the rs1183616 SNP was no longer significant (p=0.3). This suggests that the association is a single effect driven by the rs7957619 SNP. This SNP lies within exon 3 of the *MVK* gene and is a Serine to Asparagine substitution at position 52. Odds ratio plots for the rs7957619 and the rs1183616 SNPs in the UK JIA subtypes are shown in Figure 2a and b.

None of the 16 SNPs studied across the *NLRP1* gene locus were found to be associated with JIA susceptibility (Additional file 1: Table S1).

Of the four significantly associated SNPs, only the two SNPs in the *MVK* gene had genotype data available in a validation cohort (US cases and controls). The SNPs in the *IL1 ligand* and the *TNFRSF1A* genes were not genotyped on the Affymetrix array and there were no proxies (r^2^>0.8) for these SNPs. Of the two SNPs in the *MVK* gene, one SNP, rs7957619, has been directly genotyped and showed significant evidence for association with US JIA (ptrend=0.04 OR 0.83 95% CI 0.7-0.99) (Table 2). However, this association is in the opposite direction to the association in UK JIA cases, where the minor allele was associated with increased risk of developing JIA. The second SNP, rs11836136, was not directly genotyped on the array but a complete proxy, rs888193, showed no association with JIA (ptrend=0.22) (Table 2).

**Table 2:** ***MVK *SNPs investigated for association in the US cohort**

**SNP**	**CHR**	**Position**	**Gene**	**Major allele (allele 1)**	**Minor allele (allele 2)**	**MAF Case**	**MAF Control**	**pHWE Control**	**Case genotype frequencies**			**Control genotype frequencies**				**Odds ratio 95% CI**
									**1/1 (%)**	**1/2 (%)**	**2/2 (%)**	**1/1 (%)**	**1/2 (%)**	**2/2 (%)**	**p-value**	
rs888193*	12	109987576	*MVK*	G	C	0.25	0.26	0.22	450	313	41	1679	1148	220	0.22	0.92
									(56.0)	(38.9)	(5.1)	(55.1)	(37.7)	(7.2)		0.81-1.05
**rs7957619**	**12**	**110013879**	***MVK***	**G**	**A**	**0.10**	**0.12**	**0.67**	**653**	**150**	**9**	**2358**	**650**	**48**	**0.04**	**0.83**
									**(80.4)**	**(18.5)**	**(1.1)**	**(77.2)**	**(21.3)**	**(1.6)**		**0.7-0.99**

* rs888193 is a perfect proxy (r^2^=1) for rs11836136.

CHR=chromosome, MAF=minor allele frequency, CI=confidence interval, pHWE=p-value for departure from Hardy-Weinburg equilibrium.

## Discussion

In this study we have utilised a large cohort of JIA cases and identified associations between UK JIA and polymorphisms in three genes: *MVK, TNFRSF1A,* and *IL1A*. These findings follow on from work performed by our group, showing associations between psoriatic JIA and other HPF syndrome genes, and work previously showing associations between SoJIA and the *IL1 ligand* and *IL1 receptor* gene clusters [7,18].

After applying a Bonferroni correction for the number of loci studied (p<0.0013), none of the loci associated with UK JIA would remain significant. However, this is a highly conservative correction method which assumes all tests are independent, as it does not take into account correlations between SNPs. We have thus taken the approach of trying to validate significant findings in an independent cohort.

For two of the four significantly associated SNPs in the UK cohort we had validation data from a US cohort. One SNP showed no association in this cohort and the other SNP showed association but in the opposite direction. The US cohort comprises JIA cases from only three ILAR subtypes: RF negative polyarthritis, persistent and extended oligoarthritis subtypes, whereas the UK cohort encompasses all seven of the ILAR JIA subtypes. Analysis of the UK dataset stratified by ILAR subtype for the rs7957619 and rs11836136 SNPs suggests the association with JIA overall is driven largely by the systemic, together with the RF positive polyarthritis and psoriatic arthritis subtypes (Figure 2a and b). Analysis with just the RF negative polyarthritis and oligoarthritis subtypes showed no association for either rs11836136 (ptrend=0.3) or rs7957619 (ptrend=0.12). Conversely, analysis of the UK dataset with all the other subtypes (systemics, RF positive polyarthritis, enthesitis related arthritis and psoriatic JIA) showed association with rs11836136 (ptrend=0.005) and rs7957619 (ptrend=0.02). Therefore, this represents evidence for subtype specific associations. This is an interesting finding in itself as it provides further genetic evidence that the SoJIA cases are different to the other subtypes. Further investigations into the genetics of individual subtypes of JIA are required however, power is a big issue as stratification by subtype inevitably leads to small sample sizes. The lack of, or weak, association of these SNPs in the oligoarthritis and RF negative polyarthritis subtypes in the initial cohort may have been due to a lack of power and so investigating another cohort of US JIA cases and controls was appropriate. This approach has strengthened the evidence that these finding represent subtype specific effects. Independent validation of the finding in SoJIA is still required in other datasets.

The *MVK* gene encodes for mevalonate kinase, an enzyme that plays a key role in steroid synthesis. It catalyses the conversion of mevalonic acid to 5-phosphomevalonic acid, and mutations in this gene are responsible for both the hyper-IgD syndrome (HIDS), and the more severe mevalonic aciduria [14,23]. The pathogenesis of HIDS is not completely clear, but despite very high levels of IgD seen in this disease, it is thought this is unlikely to exert a direct pathological effect. More likely it is a reduction in levels of isoprenoids, anti-inflammatory compounds produced downstream from MVK in the steroid synthesis pathway that causes the proinflammatory phenotype seen in HIDS [14]. Experimental models have shown that lack of isoprenoids in HIDS causes an increase in the secretion of the proinflammatory cytokine IL-1β [24,25]. The rs7957619 SNP associated with UK JIA is exonic and changes an amino acid (from Serine to Asparagine). Once replication of the genetic association has been achieved functional analysis of this coding SNP should be undertaken to determine if this variation alters the quality or quantity of the resulting MVK protein and if any downstream effects on steroid synthesis results.

We also found association of a SNP close to *TNFRSF1A. TNFSR1A* encodes the p55 (type-1) TNF receptor (TNFR1), which plays a vital role in regulating the innate immune system via TNF-α signaling, resulting in downstream cytokine secretion, NF-kB activation, and apoptosis. The rs2228576 SNP lies 3’ of *TNFRSF1A,* within exon 13 of *SCNN1A*. The SNP results in a Threonine to Alanine amino acid change. The *SCNN1A* gene itself, which is primarily involved in sodium and water transport across epithelia, seems an unlikely gene to have functional relevance in JIA. However, once replicated, the ability of rs2228576 to regulate *TNFSR1A* function could be determined using chromosome conformation capture techniques [26,27]. This would have direct relevance to JIA disease pathogenesis. We found association of a SNP in the *IL1A* gene with the total UK JIA dataset. Stratification of this SNP by ILAR subtype however, again showed that this was largely driven by association in the SoJIA subtype. It is important to point out that there is almost complete overlap between the SoJIA cases studied here and by Stock *et al.*[18]. Therefore, the *IL1 ligand* and *receptor* SNP associations described with SoJIA herein are not independent validation of Stock’s findings. However, our data does provide evidence to suggest that the *IL1 ligand* and *receptor* SNP associations are subtype specific, being associated primarily with SoJIA, and possibly also with the psoriatic JIA subtype. Interestingly, there was a trend also towards significance with the *MVK* rs7957619 (p=0.08) and rs11836136 (p=0.06) SNPs with psoriatic JIA. The numbers in this subtype are small and independent replication is required however, it is intriguing that trends towards association with additional autoinflammatory disease loci and psoriatic JIA subtype are occurring, especially as we have previously reported significant association with SNPs in the autoinflammatory disease genes *NLRP3*, *NOD2*, *MEFV* and *PSTPIP1* with psoriatic JIA [7].

Magitta *et al.* previously found no association with two SNPs in *NLRP1* when 505 Norwegian JIA cases were studied [13]. We have conducted a comprehensive SNP analysis of the whole *NLRP1* gene, and its 5’ and 3’ flanking regions and found no association with UK JIA.

Although JIA is the most common rheumatic disease of childhood, it is nevertheless extremely rare, making large cohorts difficult to collect. This makes small sample sizes a continuing problem across all JIA research, especially research focused on the less common subtype presentations. Here we have used the largest cohort of JIA available in Europe, and utilised data from the Welcome Trust Case Control Consortium to provide control genotype data, to maximise study power. We have identified association to SNPs in three genes that appear to be specific with SoJIA, highlighting that there are genetic as well as clinical differences between SoJIA and the other JIA subtypes.

## Conclusions

1. We have found preliminary evidence of association of SNPs in *MVK,* 3’ of *TNFRSF1A,* and in *IL1A* genes with UK SoJIA, further establishing the genetic as well as clinical differences between SoJIA and the other JIA subtypes.

2. Further studies in other JIA cohorts should be performed to replicate these findings.

## Competing interests

The authors’ declare that they have no competing interests.

## Authors’ contributions

AH carried out genotyping, analysis of the data and co-wrote the manuscript. PH and CJS contributed to the genotyping. CAPS, BSPAR Study Group, WT & JP provided UK samples. TGD & AVR helped plan the study. RPD planned the study design, contributed to the analysis of the data and co-wrote the manuscript. All authors read and approved the final manuscript.

## Authors’ information

Childhood arthritis prospective study (CAPS):

Eileen Baildam, Lynsey Brown, Joanne Buckley, Alice Chieng, Joyce Davidson, Michael Eltringham, Helen Foster, Mark Friswell, Janet Gardner-Medwin, Paul Gilbert, Kimme Hyrich, Julie Jones, Sham Lal, Mark Lay, Carol Lydon, Alexandra Meijer, Vicki Price, Jane Sim, Maureen Todd, Peter Ward, Lucy Wedderburn.

British Society of Paediatric and Adolescent Rheumatology (BSPAR) study group:

M. Abinum, MD, M. Becker, MD, A. Bell, MD, A. Craft, MD, E. Crawley, MD, J. David, MD, H. Foster, MD, J. Gardener-Medwin, MD, J. Griffin, MD, A. Hall, MD, M. Hall, MD, A. Herrick, MD, P. Hollingworth, MD, L. Holt, MD, S. Jones, MD, G. Pountain, MD, C. Ryder, MD, T. Southwood, MD, I. Stewart, MD, H. Venning. L. Wedderburn, MD, P. Woo, MD, and S. Wyatt, MD.

## Supplementary Material

Additional file 1: Table S1 Association analysis of all SNPs studied. **Table S2**. Association analysis for all SNPs in systemic onset, persistent oligoarthritis and extended oligoarthritis subtypes. **Table S3**. Association analysis for all SNPs in RF –ve polyarthritis, RF +ve polyarthritis and enthesitis related JIA subtypes. **Table S4**. Association analysis for all SNPs in Psoriatic JIA subtype. **Table S5**. Association analysis in JIA cases (not including systemic JIA cases) and controls for SNPs in *IL1 ligand* cluster and *IL1 receptor.* Click here for file

## References

[B1] Petty RE Southwood TR Manners P Baum J Glass DN Goldenberg J He X Maldonado-Cocco J Orozco-Alcala J Prieur AM International league of associations for rheumatology classification of juvenile idiopathic arthritis: second revision, Edmonton, 2001 J Rheumatol 2004 31 390 392 14760812

[B2] Lamb R Thomson W Ogilvie E Donn R Wnt-1-inducible signaling pathway protein 3 and susceptibility to juvenile idiopathic arthritis Arthritis Rheum 2005 52 3548 3553 10.1002/art.21392 16255026

[B3] Lamb R Thomson W Ogilvie EM Donn R Positive association of SLC26A2 gene polymorphisms with susceptibility to systemic-onset juvenile idiopathic arthritis Arthritis Rheum 2007 56 1286 1291 10.1002/art.22444 17393463

[B4] Ji W Foo JN O'Roak BJ Zhao H Larson MG Simon DB Newton-Cheh C State MW Levy D Lifton RP Rare independent mutations in renal salt handling genes contribute to blood pressure variation Nat Genet 2008 40 592 599 10.1038/ng.118 18391953 PMC3766631

[B5] Tobin MD Tomaszewski M Braund PS Hajat C Raleigh SM Palmer TM Caulfield M Burton PR Samani NJ Common variants in genes underlying monogenic hypertension and hypotension and blood pressure in the general population Hypertension 2008 51 1658 1664 10.1161/HYPERTENSIONAHA.108.112664 18443236

[B6] Voight BF Scott LJ Steinthorsdottir V Morris AP Dina C Welch RP Zeggini E Huth C Aulchenko YS Thorleifsson G Twelve type 2 diabetes susceptibility loci identified through large-scale association analysis Nat Genet 2010 42 579 589 10.1038/ng.609 20581827 PMC3080658

[B7] Day TG Ramanan AV Hinks A Lamb R Packham J Wise C Punaro M Donn RP Autoinflammatory genes and susceptibility to psoriatic juvenile idiopathic arthritis Arthritis Rheum 2008 58 2142 2146 10.1002/art.23604 18576390 PMC2688675

[B8] Simon A van der Meer JW Pathogenesis of familial periodic fever syndromes or hereditary autoinflammatory syndromes Am J Physiol Regul Integr Comp Physiol 2007 292 R86 R98 16931648 10.1152/ajpregu.00504.2006

[B9] van der Hilst JC Frenkel J Hyperimmunoglobulin D syndrome in childhood Curr Rheumatol Rep 2010 12 101 107 10.1007/s11926-010-0086-1 20425018

[B10] Schoindre Y Feydy A Giraudet-Lequintrec JS Kahan A Allanore Y TNF receptor-associated periodic syndrome (TRAPS): a new cause of joint destruction? Joint Bone Spine 2009 76 567 569 10.1016/j.jbspin.2009.08.002 19796978

[B11] Martinon F Tschopp J Inflammatory caspases: linking an intracellular innate immune system to autoinflammatory diseases Cell 2004 117 561 574 10.1016/j.cell.2004.05.004 15163405

[B12] Jin Y Mailloux CM Gowan K Riccardi SL LaBerge G Bennett DC Fain PR Spritz RA NALP1 In vitiligo-associated multiple autoimmune disease N Engl J Med 2007 356 1216 1225 10.1056/NEJMoa061592 17377159

[B13] Magitta NF Boe Wolff AS Johansson S Skinningsrud B Lie BA Myhr KM Undlien DE Joner G Njolstad PR Kvien TK A coding polymorphism in NALP1 confers risk for autoimmune Addison's disease and type 1 diabetes Genes Immun 2009 10 120 124 10.1038/gene.2008.85 18946481

[B14] Stojanov S Kastner DL Familial autoinflammatory diseases: genetics, pathogenesis and treatment Curr Opin Rheumatol 2005 17 586 599 10.1097/bor.0000174210.78449.6b 16093838

[B15] Allantaz F Chaussabel D Stichweh D Bennett L Allman W Mejias A Ardura M Chung W Smith E Wise C Blood leukocyte microarrays to diagnose systemic onset juvenile idiopathic arthritis and follow the response to IL-1 blockade J Exp Med 2007 204 2131 2144 10.1084/jem.20070070 17724127 PMC2118700

[B16] Barnes MG Grom AA Thompson SD Griffin TA Pavlidis P Itert L Fall N Sowders DP Hinze CH Aronow BJ Subtype-specific peripheral blood gene expression profiles in recent-onset juvenile idiopathic arthritis Arthritis Rheum 2009 60 2102 2112 10.1002/art.24601 19565513 PMC2782469

[B17] Gattorno M Piccini A Lasiglie D Tassi S Brisca G Carta S Delfino L Ferlito F Pelagatti MA Caroli F The pattern of response to anti-interleukin-1 treatment distinguishes two subsets of patients with systemic-onset juvenile idiopathic arthritis Arthritis Rheum 2008 58 1505 1515 10.1002/art.23437 18438814

[B18] Stock CJ Ogilvie EM Samuel JM Fife M Lewis CM Woo P Comprehensive association study of genetic variants in the IL-1 gene family in systemic juvenile idiopathic arthritis Genes Immun 2008 9 349 357 10.1038/gene.2008.24 18418395

[B19] Packham JC Hall MA Long-term follow-up of 246 adults with juvenile idiopathic arthritis: functional outcome Rheumatology (Oxford) 2002 41 1428 1435 10.1093/rheumatology/41.12.1428 12468825

[B20] Adib N Hyrich K Thornton J Lunt M Davidson J Gardner-Medwin J Foster H Baildam E Wedderburn L Thomson W Association between duration of symptoms and severity of disease at first presentation to paediatric rheumatology: results from the childhood arthritis prospective study Rheumatology (Oxford) 2008 47 991 995 10.1093/rheumatology/ken085 18417527 PMC2430218

[B21] Gauderman WJ Sample size requirements for matched case–control studies of gene-environment interaction Stat Med 2002 21 35 50 10.1002/sim.973 11782049

[B22] Purcell S Neale B Todd-Brown K Thomas L Ferreira MA Bender D Maller J Sklar P de Bakker PI Daly MJ PLINK: a tool set for whole-genome association and population-based linkage analyses Am J Hum Genet 2007 81 559 575 10.1086/519795 17701901 PMC1950838

[B23] Ryan JG Goldbach-Mansky R The spectrum of autoinflammatory diseases: recent bench to bedside observations Curr Opin Rheumatol 2008 20 66 75 10.1097/BOR.0b013e3282f1bf4b 18281860 PMC4565798

[B24] Frenkel J Rijkers GT Mandey SH Buurman SW Houten SM Wanders RJ Waterham HR Kuis W Lack of isoprenoid products raises ex vivo interleukin-1beta secretion in hyperimmunoglobulinemia D and periodic fever syndrome Arthritis Rheum 2002 46 2794 2803 10.1002/art.10550 12384940

[B25] Normand S Massonnet B Delwail A Favot L Cuisset L Grateau G Morel F Silvain C Lecron JC Specific increase in caspase-1 activity and secretion of IL-1 family cytokines: a putative link between mevalonate kinase deficiency and inflammation Eur Cytokine Netw 2009 20 101 107 19825518 10.1684/ecn.2009.0163

[B26] Davison LJ Wallace C Cooper JD Cope NF Wilson NK Smyth DJ Howson JM Saleh N Al-Jeffery A Angus KL Long-range DNA looping and gene expression analyses identify DEXI as an autoimmune disease candidate gene Hum Mol Genet 2012 21 322 333 10.1093/hmg/ddr468 21989056 PMC3276289

[B27] Papantonis A Kohro T Baboo S Larkin JD Deng B Short P Tsutsumi S Taylor S Kanki Y Kobayashi M TNFalpha signals through specialized factories where responsive coding and miRNA genes are transcribed EMBO J 2012 31 4404 4414 10.1038/emboj.2012.288 23103767 PMC3512387

