# Supplementary material for: Autoinflammatory gene polymorphisms and susceptibility to UK juvenile idiopathic arthritis
Source: Pediatr Rheumatol Online J. 2013 Apr 2;11:14. doi: 10.1186/1546-0096-11-14 (PMC3621775; doi:10.1186/1546-0096-11-14)
Supplement: Additional file 1: Table S1 — Association analysis of all SNPs studied. Table S2. Association analysis for all SNPs in systemic onset, persistent oligoarthritis and extended oligoarthritis subtypes. Table S3. Association analysis for all SNPs in RF –ve polyarthritis, RF +ve polyarthritis and enthesitis related JIA subtypes. Table S4. Association analysis for all SNPs in Psoriatic JIA subtype. Table S5. Association analysis in JIA cases (not including systemic JIA cases) and controls for SNPs in IL1 ligand cluster and IL1 receptor. [file 1546-0096-11-14-S1.doc]

Supplementary Table 1 Association analysis of all SNPs studied.

| SNP | CHR | Position | Gene | Major  allele  (allele 1) | Minor  allele  (allele 2) | MAF  Case | MAF  Control | pHWE  Control | Case genotype frequencies | | | Control genotype frequencies | | | p-value | Odds ratio  95% CI |
| --- | --- | --- | --- | --- | --- | --- | --- | --- | --- | --- | --- | --- | --- | --- | --- | --- |
|  |  |  |  |  |  |  |  |  | 1/1  (%) | 1/2  (%) | 2/2  (%) | 1/1  (%) | 1/2  (%) | 2/2  (%) |  |  |
| rs2190360 | 2 | 102576098 | *IL1R* | A | G | 0.36 | 0.35 | 0.76 | 388  (39.6) | 473  (48.2) | 120  (12.2) | 2170  (41.7) | 2386  (45.9) | 643  (12.4) | 0.38 | 1.05  0.95-1.16 |
| rs6712572 | 2 | 113520678 | *IL1 ligand* | G | T | 0.53 | 0.5 | 0.39 | 251  (27) | 474  (51) | 204  (22) | 191  (24.2) | 408  (51.6) | 191  (24.2) | 0.14 | 1.11  0.97-1.27 |
| **rs2071374** | **2** | **113537352** | *IL1 ligand* | **A** | **C** | **0.30** | **0.27** | **0.67** | **453**  **(48.7)** | **392**  **(42.2)** | **85**  **(9.1)** | **2751**  **(53.0)** | **2067**  **(39.8)** | **376**  **(7.2)** | **0.006** | **1.16**  **1.04-1.30** |
| rs1688075 | 2 | 113858196 | *IL1 ligand* | A | C | 0.08 | 0.07 | 0.03 | 793  (85.3) | 130  (14) | 7  (0.8) | 693  (87.30 | 93  (11.7) | 8  (1) | 0.34 | 1.14  0.88-1.47 |
| rs1860545 | 12 | 6446777 | *TNFRSF1A* | G | A | 0.39 | 0.40 | 0.27 | 367  (37.3) | 471  (47.8) | 147  (14.9) | 1871  (36.0) | 2528  (48.6) | 800  (15.4) | 0.47 | 0.96  0.87-1.06 |
| rs4149579 | 12 | 6447357 | *TNFRSF1A* | G | A | 0.09 | 0.08 | 0.20 | 775  (83.2) | 147  (15.8) | 9  (1.0) | 4377  (84.2) | 776  (14.9) | 43  (0.8) | 0.42 | 1.08  0.90-1.28 |
| rs4149578 | 12 | 6447437 | *TNFRSF1A* | G | A | 0.10 | 0.09 | 0.33 | 767  (82.6) | 145  (15.6) | 17  (1.8) | 4266  (82.1) | 894  (17.2) | 39  (0.8) | 0.70 | 1.03  0.87-1.22 |
| rs4149577 | 12 | 6447522 | *TNFRSF1A* | T | C | 0.49 | 0.49 | 0.56 | 247  (26.5) | 459  (49.3) | 225  (24.2) | 1373  (26.4) | 2577  (49.6) | 1249  (24.0) | 0.99 | 1.00  0.91-1.10 |
| rs4149576 | 12 | 6449115 | *TNFRSF1A* | G | A | 0.41 | 0.42 | 0.53 | 338  (34.3) | 487  (49.4) | 160  (16.2) | 1747  (33.6) | 2552  (49.1) | 898  (17.3) | 0.47 | 0.96  0.87-1.06 |
| rs4149573 | 12 | 6449384 | *TNFRSF1A* | G | C | 0.09 | 0.09 | 0.80 | 821  (83.2) | 152  (15.4) | 14  (1.4) | 4318  (83.1) | 834  (16.1) | 42  (0.8) | 0.69 | 1.04  0.88-1.22 |
| rs767455 | 12 | 6450945 | *TNFRSF1A* | T | C | 0.41 | 0.42 | 0.51 | 331  (33.5) | 493  (49.9) | 163  (16.5) | 1736  (33.4) | 2554  (49.2) | 904  (17.4) | 0.68 | 0.98  0.89-1.08 |
| rs4149570 | 12 | 6451590 | *TNFRSF1A* | G | T | 0.41 | 0.42 | 0.65 | 315  (33.9) | 471  (50.7) | 143  (15.4) | 1745  (33.6) | 2515  (48.4) | 931  (17.9) | 0.25 | 0.94  0.85-1.04 |
| rs11064145 | 12 | 6455098 | *TNFRSF1A* | T | G | 0.43 | 0.43 | 0.41 | 309  (31.3) | 502  (50.9) | 176  (17.8) | 1649  (31.7) | 2585  (49.7) | 966  (18.6) | 0.89 | 0.99  0.90-1.09 |
| **rs2228576** | **12** | **6457062** | *TNFRSF1A* | **G** | **A** | **0.33** | **0.36** | **0.43** | **414**  **(44.7)** | **419**  **(45.2)** | **94**  **(10.1)** | **2136**  **(41.4)** | **2349**  **(45.5)** | **678**  **(13.1)** | **0.009** | **0.87**  **0.78-0.97** |
| rs3764875 | 12 | 6458084 | *TNFRSF1A* | A | G | 0.36 | 0.35 | 0.65 | 386  (41.5) | 426  (45.8) | 118  (12.7) | 2174  (41.8) | 2363  (45.5) | 660  (12.7) | 0.90 | 1.01  0.91-1.12 |
| **rs11836136** | **12** | **110006512** | ***MVK*** | **A** | **G** | **0.29** | **0.26** | **1.00** | **460**  **(50.3)** | **380**  **(41.6)** | **74**  **(8.1)** | **2857**  **(55.0)** | **1992**  **(38.3)** | **347**  **(6.7)** | **0.006** | **1.17**  **1.04-1.30** |
| **rs7957619** | **12** | **110013879** | ***MVK*** | **G** | **A** | **0.14** | **0.11** | **0.33** | **699**  **(75.2)** | **210**  **(22.6)** | **21**  **(2.3)** | **4099**  **(78.8)** | **1027**  **(19.8)** | **73**  **(1.4)** | **0.005** | **1.23**  **1.07-1.43** |
| rs11067376 | 12 | 110027795 | *MVK* | A | G | 0.37 | 0.36 | 0.07 | 352  (42.3) | 352  (42.3) | 129  (15.5) | 2071  (39.8) | 2469  (47.5) | 659  (12.7) | 0.88 | 1.01  0.91-1.12 |
| rs2270374 | 12 | 110029186 | *MVK* | C | T | 0.17 | 0.18 | 0.43 | 675  (68.5) | 280  (28.4) | 31  (3.1) | 3448  (66.3) | 1582  (30.4) | 168  (3.2) | 0.24 | 0.93  0.82-1.05 |
| rs17183465 | 12 | 110029750 | *MVK* | T | A | 0.18 | 0.18 | 0.53 | 673  (68.2) | 282  (28.6) | 32  (3.2) | 3569  (66.3) | 1635  (30.4) | 176  (3.3) | 0.32 | 0.94  0.83-1.06 |
| rs10161126 | 12 | 110042348 | *MVK* | G | A | 0.45 | 0.47 | 0.11 | 309  (31.4) | 456  (46.3) | 220  (22.3) | 1423  (27.4) | 2649  (51.0) | 1127  (21.7) | 0.17 | 0.94  0.85-1.03 |
| rs1541597 | 12 | 110044918 | *MVK* | C | A | 0.22 | 0.20 | 0.90 | 568  (61.0) | 322  (34.6) | 41  (4.4) | 3327  (64.0) | 1664  (32.0) | 205  (3.9) | 0.08 | 1.11  0.99-1.25 |
| rs5862 | 17 | 5402957 | *NLRP1* | A | G | 0.47 | 0.47 | 0.70 | 206  (27.8) | 376  (50.7) | 160  (21.6) | 1464  (28.2) | 2603  (50.1) | 1131  (21.8) | 0.94 | 1.00  0.90-1.12 |
| rs8079034 | 17 | 5412361 | *NLRP1* | C | T | 0.19 | 0.20 | 0.93 | 490  (65.6) | 226  (30.3) | 31  (4.1) | 3349  (64.5) | 1647  (31.7) | 200  (3.8) | 0.70 | 0.97  0.85-1.12 |
| rs12602960 | 17 | 5414326 | *NLRP1* | G | T | 0.26 | 0.26 | 0.10 | 552  (56.0) | 362  (36.7) | 72  (7.3) | 2938  (54.6) | 2042  (38.0) | 398  (7.4) | 0.51 | 0.96  0.86-1.08 |
| rs4790267 | 17 | 5415287 | *NLRP1* | G | A | 0.39 | 0.40 | 0.79 | 371  (37.7) | 462  (47.0) | 151  (15.3) | 1869  (36.0) | 2497  (48.1) | 820  (15.8) | 0.38 | 0.96  0.87-1.06 |
| rs9911319 | 17 | 5415623 | *NLRP1* | C | T | 0.45 | 0.45 | 0.93 | 225  (30.2) | 374  (50.1) | 147  (19.7) | 1592  (30.6) | 2573  (49.5) | 1034  (19.9) | 0.92 | 1.01  0.90-1.12 |
| rs9900356 | 17 | 5419278 | *NLRP1* | T | C | 0.21 | 0.22 | 0.42 | 467  (62.6) | 239  (32.0) | 40  (5.4) | 3145  (60.5) | 1783  (34.3) | 269  (5.2) | 0.41 | 0.95  0.83-1.08 |
| rs6502867 | 17 | 5420328 | *NLRP1* | T | C | 0.23 | 0.24 | 0.54 | 416  (58.9) | 249  (35.3) | 41  (5.8) | 3038  (58.4) | 1863  (35.8) | 299  (5.8) | 0.85 | 0.99  0.87-1.13 |
| rs12937224 | 17 | 5424042 | *NLRP1* | T | C | 0.45 | 0.45 | 0.28 | 294  (30.1) | 479  (49.0) | 204  (20.9) | 1610  (29.9) | 2702  (50.2) | 1068  (19.9) | 0.72 | 1.02  0.92-1.12 |
| rs2301582 | 17 | 5436263 | *NLRP1* | C | T | 0.39 | 0.39 | 0.75 | 284  (38.1) | 345  (46.2) | 117  (15.7) | 1949  (37.5) | 2458  (47.3) | 790  (15.2) | 0.98 | 1.00  0.89-1.12 |
| rs11652907 | 17 | 5449665 | *NLRP1* | T | C | 0.17 | 0.16 | 0.64 | 515  (68.9) | 206  (27.6) | 26  (3.5) | 3676  (70.7) | 1386  (26.7) | 137  (2.6) | 0.20 | 1.10  0.95-1.27 |
| rs4790777 | 17 | 5451162 | *NLRP1* | T | C | 0.39 | 0.39 | 0.77 | 284  (38.1) | 345  (46.2) | 117  (15.7) | 2037  (37.9) | 2537  (47.2) | 803  (14.9) | 0.83 | 1.01  0.91-1.13 |
| rs12952888 | 17 | 5458824 | *NLRP1* | C | T | 0.05 | 0.05 | 0.58 | 670  (89.7) | 76  (10.2) | 1  (0.1) | 1555  (90.8) | 156  (9.1) | 2  (0.1) | 0.40 | 1.12  0.85-1.48 |
| rs961826 | 17 | 5483937 | *NLRP1* | G | T | 0.47 | 0.46 | 0.98 | 269  (27.5) | 497  (50.8) | 212  (21.7) | 1538  (29.6) | 2577  (49.6) | 1081  (20.8) | 0.23 | 1.06  0.96-1.17 |
| rs11078575 | 17 | 5485785 | *NLRP1* | A | G | 0.48 | 0.46 | 0.91 | 203  (27.4) | 369  (49.7) | 170  (22.9) | 1531  (29.4) | 2585  (49.7) | 1083  (20.8) | 0.13 | 1.09  0.98-1.21 |
| rs871928 | 17 | 5486800 | *NLRP1* | G | A | 0.19 | 0.17 | 0.77 | 495  (66.5) | 222  (29.8) | 27  (3.6) | 3547  (68.2) | 1490  (28.7) | 161  (3.1) | 0.29 | 1.08  0.94-1.24 |
| rs925596 | 17 | 5488099 | *NLRP1* | A | G | 0.09 | 0.11 | 0.57 | 614  (82.7) | 117  (15.8) | 11  (1.5) | 4121  (79.3) | 1012  (19.5) | 67  (1.3) | 0.06 | 0.83  0.69-1.00 |

CHR=chromosome, MAF=minor allele frequency, CI=confidence interval, pHWE= p-value for departure from Hardy-Weinburg equilibrium

Supplementary Table 2 Association analysis for all SNPs in systemic onset, persistent oligoarthritis and extended oligoarthritis subtypes

|  |  |  |  | Systemic onset JIA | |  |  | Persistent oligoarthritis | |  |  | Extended oligoarthritis | |  |  |
| --- | --- | --- | --- | --- | --- | --- | --- | --- | --- | --- | --- | --- | --- | --- | --- |
| SNP | CHR | Position | Gene | MAF cases | MAF Controls | p-value | OR (95% CI) | MAF cases | MAF Controls | p-value | OR (95% CI) | MAF cases | MAF Controls | p-value | OR (95% CI) |
| rs2190360 | 2 | 102576098 | il1r | 0.42 | 0.35 | 0.02 | 1.34 (1.05-1.7) | 0.36 | 0.35 | 0.76 | 1.03 (0.85-1.24) | 0.38 | 0.35 | 0.45 | 1.1 (0.85-1.43) |
| rs6712572 | 2 | 113520678 | il1ligand | 0.58 | 0.5 | 0.009 | 1.39 (1.08-1.79) | 0.51 | 0.5 | 0.57 | 1.06 (0.86-1.29) | 0.48 | 0.5 | 0.64 | 0.94 (0.72-1.22) |
| rs2071374 | 2 | 113537352 | il1ligand | 0.36 | 0.27 | 0.001 | 1.5 (1.16-1.92) | 0.3 | 0.27 | 0.24 | 1.13 (0.92-1.38) | 0.27 | 0.27 | 0.98 | 1 (0.75-1.33) |
| rs1688075 | 2 | 113858196 | il1ligand | 0.09 | 0.07 | 0.13 | 1.42 (0.92-2.19) | 0.07 | 0.07 | 0.69 | 1.08 (0.74-1.6) | 0.1 | 0.07 | 0.09 | 1.48 (0.94-2.34) |
| rs1860545 | 12 | 6446777 | TNFRSF1A | 0.4 | 0.4 | 0.88 | 1.02 (0.8-1.3) | 0.4 | 0.4 | 0.95 | 1.01 (0.84-1.21) | 0.35 | 0.4 | 0.11 | 0.81 (0.62-1.06) |
| rs4149579 | 12 | 6447357 | TNFRSF1A | 0.08 | 0.08 | 0.69 | 0.91 (0.58-1.43) | 0.1 | 0.08 | 0.32 | 1.17 (0.86-1.6) | 0.07 | 0.08 | 0.61 | 0.88 (0.54-1.43) |
| rs4149578 | 12 | 6447437 | TNFRSF1A | 0.07 | 0.09 | 0.18 | 0.73 (0.45-1.17) | 0.08 | 0.09 | 0.29 | 0.83 (0.59-1.17) | 0.14 | 0.09 | 0.01 | 1.57 (1.09-2.27) |
| rs4149577 | 12 | 6447522 | TNFRSF1A | 0.48 | 0.49 | 0.75 | 0.96 (0.76-1.22) | 0.5 | 0.49 | 0.67 | 1.04 (0.87-1.25) | 0.48 | 0.49 | 0.89 | 0.98 (0.76-1.27) |
| rs4149576 | 12 | 6449115 | TNFRSF1A | 0.43 | 0.42 | 0.79 | 1.03 (0.81-1.31) | 0.43 | 0.42 | 0.76 | 1.03 (0.86-1.23) | 0.37 | 0.42 | 0.15 | 0.83 (0.64-1.07) |
| rs4149573 | 12 | 6449384 | TNFRSF1A | 0.07 | 0.09 | 0.4 | 0.82 (0.53-1.29) | 0.08 | 0.09 | 0.57 | 0.91 (0.65-1.27) | 0.12 | 0.09 | 0.14 | 1.34 (0.91-1.99) |
| rs767455 | 12 | 6450945 | TNFRSF1A | 0.43 | 0.42 | 0.66 | 1.06 (0.83-1.34) | 0.43 | 0.42 | 0.62 | 1.05 (0.87-1.26) | 0.38 | 0.42 | 0.21 | 0.85 (0.66-1.1) |
| rs4149570 | 12 | 6451590 | TNFRSF1A | 0.39 | 0.42 | 0.38 | 0.9 (0.7-1.14) | 0.4 | 0.42 | 0.4 | 0.92 (0.77-1.11) | 0.43 | 0.42 | 0.89 | 1.02 (0.79-1.32) |
| rs11064145 | 12 | 6455098 | TNFRSF1A | 0.44 | 0.43 | 0.75 | 1.04 (0.82-1.32) | 0.45 | 0.43 | 0.4 | 1.08 (0.9-1.3) | 0.41 | 0.43 | 0.49 | 0.92 (0.71-1.18) |
| rs2228576 | 12 | 6457062 | TNFRSF1A | 0.32 | 0.36 | 0.16 | 0.83 (0.64-1.08) | 0.32 | 0.36 | 0.1 | 0.85 (0.7-1.03) | 0.36 | 0.36 | 0.94 | 0.99 (0.76-1.29) |
| rs3764875 | 12 | 6458084 | TNFRSF1A | 0.35 | 0.35 | 0.92 | 0.99 (0.77-1.27) | 0.34 | 0.35 | 0.51 | 0.94 (0.77-1.14) | 0.33 | 0.35 | 0.47 | 0.91 (0.69-1.19) |
| rs11836136 | 12 | 110006512 | MVK | 0.32 | 0.26 | 0.03 | 1.34 (1.03-1.74) | 0.28 | 0.26 | 0.33 | 1.11 (0.9-1.36) | 0.25 | 0.26 | 0.88 | 0.98 (0.73-1.31) |
| rs7957619 | 12 | 110013879 | MVK | 0.14 | 0.11 | 0.14 | 1.29 (0.92-1.83) | 0.14 | 0.11 | 0.13 | 1.23 (0.94-1.61) | 0.11 | 0.11 | 0.76 | 0.94 (0.62-1.42) |
| rs11067376 | 12 | 110027795 | MVK | 0.33 | 0.36 | 0.31 | 0.87 (0.67-1.14) | 0.39 | 0.36 | 0.38 | 1.09 (0.89-1.34) | 0.36 | 0.36 | 0.92 | 0.99 (0.75-1.29) |
| rs2270374 | 12 | 110029186 | MVK | 0.15 | 0.18 | 0.13 | 0.77 (0.56-1.08) | 0.2 | 0.18 | 0.31 | 1.12 (0.9-1.41) | 0.19 | 0.18 | 0.93 | 1.01 (0.74-1.4) |
| rs17183465 | 12 | 110029750 | MVK | 0.15 | 0.18 | 0.15 | 0.79 (0.57-1.09) | 0.21 | 0.18 | 0.18 | 1.16 (0.93-1.45) | 0.18 | 0.18 | 0.93 | 0.99 (0.71-1.36) |
| rs10161126 | 12 | 110042348 | MVK | 0.45 | 0.47 | 0.48 | 0.92 (0.72-1.17) | 0.44 | 0.47 | 0.16 | 0.88 (0.73-1.05) | 0.46 | 0.47 | 0.72 | 0.96 (0.74-1.23) |
| rs1541597 | 12 | 110044918 | MVK | 0.22 | 0.2 | 0.38 | 1.14 (0.85-1.52) | 0.22 | 0.2 | 0.25 | 1.14 (0.91-1.42) | 0.19 | 0.2 | 0.79 | 0.96 (0.69-1.32) |
| rs5862 | 17 | 5402957 | NLRP1 | 0.49 | 0.47 | 0.56 | 1.07 (0.85-1.36) | 0.47 | 0.47 | 0.85 | 1.02 (0.83-1.26) | 0.5 | 0.47 | 0.34 | 1.14 (0.87-1.48) |
| rs8079034 | 17 | 5412361 | NLRP1 | 0.18 | 0.2 | 0.6 | 0.92 (0.68-1.25) | 0.19 | 0.2 | 0.85 | 0.97 (0.75-1.27) | 0.19 | 0.2 | 0.83 | 0.96 (0.69-1.34) |
| rs12602960 | 17 | 5414326 | NLRP1 | 0.26 | 0.26 | 0.8 | 0.97 (0.74-1.26) | 0.27 | 0.26 | 0.95 | 1.01 (0.82-1.24) | 0.25 | 0.26 | 0.63 | 0.93 (0.7-1.24) |
| rs4790267 | 17 | 5415287 | NLRP1 | 0.41 | 0.4 | 0.66 | 1.06 (0.83-1.34) | 0.38 | 0.4 | 0.42 | 0.93 (0.77-1.12) | 0.44 | 0.4 | 0.15 | 1.2 (0.93-1.55) |
| rs9911319 | 17 | 5415623 | NLRP1 | 0.48 | 0.45 | 0.34 | 1.12 (0.88-1.42) | 0.43 | 0.45 | 0.52 | 0.93 (0.76-1.15) | 0.5 | 0.45 | 0.11 | 1.24 (0.96-1.61) |
| rs9900356 | 17 | 5419278 | NLRP1 | 0.2 | 0.22 | 0.26 | 0.84 (0.63-1.14) | 0.21 | 0.22 | 0.56 | 0.93 (0.72-1.2) | 0.21 | 0.22 | 0.6 | 0.92 (0.67-1.27) |
| rs6502867 | 17 | 5420328 | NLRP1 | 0.24 | 0.24 | 0.96 | 0.99 (0.75-1.32) | 0.23 | 0.24 | 0.78 | 0.96 (0.74-1.25) | 0.22 | 0.24 | 0.63 | 0.92 (0.67-1.27) |
| rs12937224 | 17 | 5424042 | NLRP1 | 0.45 | 0.45 | 0.92 | 0.99 (0.78-1.25) | 0.44 | 0.45 | 0.64 | 0.96 (0.8-1.15) | 0.41 | 0.45 | 0.23 | 0.86 (0.66-1.1) |
| rs2301582 | 17 | 5436263 | NLRP1 | 0.38 | 0.39 | 0.76 | 0.96 (0.75-1.23) | 0.38 | 0.39 | 0.74 | 0.96 (0.78-1.2) | 0.37 | 0.39 | 0.47 | 0.91 (0.69-1.19) |
| rs11652907 | 17 | 5449665 | NLRP1 | 0.2 | 0.16 | 0.08 | 1.3 (0.97-1.76) | 0.17 | 0.16 | 0.65 | 1.07 (0.81-1.41) | 0.19 | 0.16 | 0.2 | 1.25 (0.89-1.74) |
| rs4790777 | 17 | 5451162 | NLRP1 | 0.38 | 0.39 | 0.73 | 0.96 (0.75-1.22) | 0.39 | 0.39 | 0.82 | 1.03 (0.83-1.27) | 0.36 | 0.39 | 0.45 | 0.9 (0.69-1.18) |
| rs12952888 | 17 | 5458824 | NLRP1 | 0.07 | 0.05 | 0.11 | 1.48 (0.9-2.41) | 0.05 | 0.05 | 0.85 | 1.05 (0.64-1.73) | 0.03 | 0.05 | 0.4 | 0.73 (0.36-1.52) |
| rs961826 | 17 | 5483937 | NLRP1 | 0.49 | 0.46 | 0.32 | 1.13 (0.89-1.43) | 0.47 | 0.46 | 0.69 | 1.04 (0.87-1.25) | 0.44 | 0.46 | 0.71 | 0.95 (0.74-1.23) |
| rs11078575 | 17 | 5485785 | NLRP1 | 0.5 | 0.46 | 0.19 | 1.17 (0.92-1.49) | 0.48 | 0.46 | 0.42 | 1.09 (0.88-1.34) | 0.44 | 0.46 | 0.68 | 0.95 (0.73-1.23) |
| rs871928 | 17 | 5486800 | NLRP1 | 0.21 | 0.17 | 0.17 | 1.23 (0.91-1.64) | 0.18 | 0.17 | 0.77 | 1.04 (0.79-1.37) | 0.21 | 0.17 | 0.16 | 1.26 (0.92-1.74) |
| rs925596 | 17 | 5488099 | NLRP1 | 0.08 | 0.11 | 0.1 | 0.69 (0.44-1.07) | 0.1 | 0.11 | 0.63 | 0.92 (0.65-1.3) | 0.09 | 0.11 | 0.29 | 0.78 (0.49-1.23) |

CHR=Chromosome, MAF=minor allele frequency, OR=Odds ratio, CI=confidence interval

Supplementary Table 3 Association analysis for all SNPs in RF –ve polyarthritis, RF +ve polyarthritis and enthesitis related JIA subtypes

|  |  |  |  | RF Neg polyarthritis | |  |  | RF pos polyarthritis | |  |  | Enthesitis related JIA | |  |  |
| --- | --- | --- | --- | --- | --- | --- | --- | --- | --- | --- | --- | --- | --- | --- | --- |
| SNP | CHR | Position | Gene | MAF cases | MAF Controls | p-value | OR (95% CI) | MAF cases | MAF Controls | p-value | OR (95% CI) | MAF cases | MAF Controls | p-value | OR (95% CI) |
| rs2190360 | 2 | 102576098 | il1r | 0.34 | 0.35 | 0.73 | 0.96 (0.77-1.2) | 0.29 | 0.35 | 0.19 | 0.76 (0.5-1.15) | 0.31 | 0.35 | 0.41 | 0.84 (0.55-1.28) |
| rs6712572 | 2 | 113520678 | il1ligand | 0.52 | 0.5 | 0.56 | 1.07 (0.85-1.34) | 0.55 | 0.5 | 0.26 | 1.24 (0.84-1.84) | 0.43 | 0.5 | 0.18 | 0.77 (0.52-1.14_ |
| rs2071374 | 2 | 113537352 | il1ligand | 0.28 | 0.27 | 0.63 | 1.06 (0.84-1.34) | 0.33 | 0.27 | 0.2 | 1.3 (0.86-1.97) | 0.25 | 0.27 | 0.64 | 0.9 (0.56-1.42) |
| rs1688075 | 2 | 113858196 | il1ligand | 0.09 | 0.07 | 0.28 | 1.27 (0.84-1.93) | 0.07 | 0.07 | 0.84 | 1.09 (0.51-2.29) | 0.06 | 0.07 | 0.65 | 0.81 (0.35-1.9) |
| rs1860545 | 12 | 6446777 | TNFRSF1A | 0.37 | 0.4 | 0.35 | 0.9 (0.73-1.12) | 0.47 | 0.4 | 0.11 | 1.36 (0.93-1.99) | 0.42 | 0.4 | 0.61 | 1.11 (0.75-1.64) |
| rs4149579 | 12 | 6447357 | TNFRSF1A | 0.11 | 0.08 | 0.09 | 1.35 (0.95-1.9) | 0.06 | 0.08 | 0.36 | 0.68 (0.3-1.55) | 0.11 | 0.08 | 0.27 | 1.43 (0.76-2.69) |
| rs4149578 | 12 | 6447437 | TNFRSF1A | 0.1 | 0.09 | 0.82 | 1.04 (0.73-1.5) | 0.09 | 0.09 | 0.81 | 0.92 (0.46-1.83) | 0.1 | 0.09 | 0.72 | 1.13 (0.58-2.18) |
| rs4149577 | 12 | 6447522 | TNFRSF1A | 0.51 | 0.49 | 0.34 | 1.11 (0.9-1.37) | 0.4 | 0.49 | 0.09 | 0.71 (0.48-1.05) | 0.45 | 0.49 | 0.43 | 0.85 (0.57-1.28) |
| rs4149576 | 12 | 6449115 | TNFRSF1A | 0.38 | 0.42 | 0.18 | 0.86 (0.7-1.07) | 0.49 | 0.42 | 0.13 | 1.34 (0.91-1.96) | 0.43 | 0.42 | 0.79 | 1.06 (0.71-1.56) |
| rs4149573 | 12 | 6449384 | TNFRSF1A | 0.09 | 0.09 | 1 | 1 (0.69-1.45) | 0.07 | 0.09 | 0.6 | 0.83 (0.4-1.7) | 0.09 | 0.09 | 1 | 1 (0.5-1.99) |
| rs767455 | 12 | 6450945 | TNFRSF1A | 0.39 | 0.42 | 0.21 | 0.87 (0.7-1.08) | 0.5 | 0.42 | 0.09 | 1.38 (0.95-2.02) | 0.43 | 0.42 | 0.81 | 1.05 (0.71-1.55) |
| rs4149570 | 12 | 6451590 | TNFRSF1A | 0.43 | 0.42 | 0.88 | 1.02 (0.82-1.26) | 0.37 | 0.42 | 0.25 | 0.79 (0.53-1.18) | 0.38 | 0.42 | 0.36 | 0.82 (0.54-1.25) |
| rs11064145 | 12 | 6455098 | TNFRSF1A | 0.4 | 0.43 | 0.2 | 0.87 (0.7-1.08) | 0.52 | 0.43 | 0.08 | 1.4 (0.96-2.05) | 0.44 | 0.43 | 0.89 | 1.03 (0.69-1.52) |
| rs2228576 | 12 | 6457062 | TNFRSF1A | 0.32 | 0.36 | 0.19 | 0.86 (0.68-1.08) | 0.31 | 0.36 | 0.28 | 0.79 (0.52-1.21) | 0.3 | 0.36 | 0.25 | 0.77 (0.5-1.2) |
| rs3764875 | 12 | 6458084 | TNFRSF1A | 0.35 | 0.35 | 1 | 1 (0.8-1.25) | 0.36 | 0.35 | 0.98 | 1.01 (0.67-1.51) | 0.38 | 0.35 | 0.67 | 1.09 (0.72-1.66) |
| rs11836136 | 12 | 110006512 | MVK | 0.28 | 0.26 | 0.39 | 1.11 (0.87-1.41) | 0.34 | 0.26 | 0.05 | 1.5 (0.99-2.26) | 0.22 | 0.26 | 0.44 | 0.83 (0.51-1.34) |
| rs7957619 | 12 | 110013879 | MVK | 0.14 | 0.11 | 0.2 | 1.23 (0.9-1.68) | 0.18 | 0.11 | 0.03 | 1.76 (1.07-2.9) | 0.08 | 0.11 | 0.37 | 0.72 (0.35-1.48) |
| rs11067376 | 12 | 110027795 | MVK | 0.36 | 0.36 | 0.97 | 1 (0.79-1.26) | 0.43 | 0.36 | 0.18 | 1.31 (0.88-1.96) | 0.31 | 0.36 | 0.26 | 0.77 (0.49-1.22) |
| rs2270374 | 12 | 110029186 | MVK | 0.18 | 0.18 | 0.92 | 0.99 (0.75-1.29) | 0.18 | 0.18 | 0.82 | 0.94 (0.57-1.55) | 0.16 | 0.18 | 0.47 | 0.82 (0.48-1.41) |
| rs17183465 | 12 | 110029750 | MVK | 0.18 | 0.18 | 0.91 | 0.98 (0.75-1.29) | 0.18 | 0.18 | 0.82 | 0.94 (0.57-1.55) | 0.16 | 0.18 | 0.47 | 0.82 (0.48-1.4) |
| rs10161126 | 12 | 110042348 | MVK | 0.47 | 0.47 | 0.94 | 0.99 (0.8-1.22) | 0.36 | 0.47 | 0.02 | 0.63 (0.43-0.94) | 0.51 | 0.47 | 0.44 | 1.17 (0.79-1.72) |
| rs1541597 | 12 | 110044918 | MVK | 0.21 | 0.2 | 0.5 | 1.09 (0.84-1.42) | 0.23 | 0.2 | 0.43 | 1.2 (0.76-1.9) | 0.18 | 0.2 | 0.58 | 0.86 (0.51-1.46) |
| rs5862 | 17 | 5402957 | NLRP1 | 0.45 | 0.47 | 0.45 | 0.91 (0.72-1.15) | 0.42 | 0.47 | 0.28 | 0.81 (0.55-1.19) | 0.49 | 0.47 | 0.7 | 1.09 (0.71-1.66) |
| rs8079034 | 17 | 5412361 | NLRP1 | 0.2 | 0.2 | 0.92 | 1.02 (0.76-1.36) | 0.13 | 0.2 | 0.08 | 0.61 (0.35-1.07) | 0.24 | 0.2 | 0.27 | 1.32 (0.8-2.16) |
| rs12602960 | 17 | 5414326 | NLRP1 | 0.27 | 0.26 | 0.96 | 1.01 (0.79-1.28) | 0.2 | 0.26 | 0.16 | 0.71 (0.45-1.14) | 0.29 | 0.26 | 0.5 | 1.16 (0.76-1.78) |
| rs4790267 | 17 | 5415287 | NLRP1 | 0.39 | 0.4 | 0.63 | 0.95 (0.76-1.18) | 0.32 | 0.4 | 0.11 | 0.72 (0.48-1.08) | 0.37 | 0.4 | 0.56 | 0.89 (0.59-1.33) |
| rs9911319 | 17 | 5415623 | NLRP1 | 0.44 | 0.45 | 0.9 | 0.98 (0.78-1.24) | 0.41 | 0.45 | 0.42 | 0.85 (0.58-1.25) | 0.47 | 0.45 | 0.73 | 1.08 (0.7-1.65) |
| rs9900356 | 17 | 5419278 | NLRP1 | 0.21 | 0.22 | 0.67 | 0.94 (0.71-1.25) | 0.23 | 0.22 | 0.84 | 1.05 (0.67-1.64) | 0.24 | 0.22 | 0.65 | 1.12 (0.69-1.84) |
| rs6502867 | 17 | 5420328 | NLRP1 | 0.24 | 0.24 | 0.86 | 1.03 (0.78-1.35) | 0.28 | 0.24 | 0.32 | 1.24 (0.81-1.9) | 0.2 | 0.24 | 0.46 | 0.82 (0.48-1.4) |
| rs12937224 | 17 | 5424042 | NLRP1 | 0.48 | 0.45 | 0.21 | 1.15 (0.93-1.41) | 0.45 | 0.45 | 0.95 | 1.01 (0.69-1.49) | 0.44 | 0.45 | 0.85 | 0.96 (0.65-1.43) |
| rs2301582 | 17 | 5436263 | NLRP1 | 0.43 | 0.39 | 0.16 | 1.18 (0.94-1.49) | 0.38 | 0.39 | 0.85 | 0.96 (0.65-1.42) | 0.37 | 0.39 | 0.76 | 0.93 (0.6-1.45) |
| rs11652907 | 17 | 5449665 | NLRP1 | 0.18 | 0.16 | 0.46 | 1.12 (0.83-1.52) | 0.19 | 0.16 | 0.47 | 1.2 (0.73-1.95) | 0.12 | 0.16 | 0.28 | 0.69 (0.36-1.34) |
| rs4790777 | 17 | 5451162 | NLRP1 | 0.43 | 0.39 | 0.16 | 1.18 (0.94-1.49) | 0.38 | 0.39 | 0.91 | 0.98 (0.66-1.44) | 0.37 | 0.39 | 0.8 | 0.95 (0.61-1.47) |
| rs12952888 | 17 | 5458824 | NLRP1 | 0.06 | 0.05 | 0.17 | 1.4 (0.86-2.29) | 0.06 | 0.05 | 0.38 | 1.41 (0.65-3.09) | 0.02 | 0.05 | 0.3 | 0.49 (0.12-1.99) |
| rs961826 | 17 | 5483937 | NLRP1 | 0.49 | 0.46 | 0.22 | 1.14 (0.92-1.41) | 0.43 | 0.46 | 0.53 | 0.89 (0.6-1.3) | 0.44 | 0.46 | 0.75 | 0.94 (0.63-1.39) |
| rs11078575 | 17 | 5485785 | NLRP1 | 0.52 | 0.46 | 0.03 | 1.29 (1.02-1.63) | 0.44 | 0.46 | 0.65 | 0.92 (0.62-1.34) | 0.46 | 0.46 | 0.89 | 1.03 (0.67-1.58) |
| rs871928 | 17 | 5486800 | NLRP1 | 0.18 | 0.17 | 0.91 | 1.02 (0.75-1.38) | 0.2 | 0.17 | 0.42 | 1.21 (0.76-1.94) | 0.15 | 0.17 | 0.57 | 0.84 (0.47-1.53) |
| rs925596 | 17 | 5488099 | NLRP1 | 0.07 | 0.11 | 0.05 | 0.65 (0.42-1.01) | 0.11 | 0.11 | 0.98 | 1.01 (0.55-1.85) | 0.12 | 0.11 | 0.86 | 1.06 (0.55-2.06) |

CHR=Chromosome, MAF=minor allele frequency, OR=Odds ratio, CI=confidence interval

Supplementary Table 4 Association analysis for all SNPs in Psoriatic JIA subtype

|  |  |  |  | Psoriatic JIA | |  |  |
| --- | --- | --- | --- | --- | --- | --- | --- |
| SNP | CHR | Position | Gene | MAF cases | MAF Controls | p-value | OR (95% CI) |
| rs2190360 | 2 | 102576098 | il1r | 0.4 | 0.35 | 0.27 | 1.22 (0.86-1.74) |
| rs6712572 | 2 | 113520678 | il1ligand | 0.54 | 0.5 | 0.4 | 1.16 (0.81-1.66) |
| rs2071374 | 2 | 113537352 | il1ligand | 0.34 | 0.27 | 0.11 | 1.36 (0.93-1.98) |
| rs1688075 | 2 | 113858196 | il1ligand | 0.05 | 0.07 | 0.31 | 0.65 (0.28-1.5) |
| rs1860545 | 12 | 6446777 | TNFRSF1A | 0.4 | 0.4 | 0.94 | 1.01 (0.71-1.44) |
| rs4149579 | 12 | 6447357 | TNFRSF1A | 0.11 | 0.08 | 0.21 | 1.43 (0.82-2.51) |
| rs4149578 | 12 | 6447437 | TNFRSF1A | 0.11 | 0.09 | 0.62 | 1.16 (0.65-2.06) |
| rs4149577 | 12 | 6447522 | TNFRSF1A | 0.48 | 0.49 | 0.92 | 0.98 (0.69-1.4) |
| rs4149576 | 12 | 6449115 | TNFRSF1A | 0.42 | 0.42 | 0.91 | 1.02 (0.72-1.45) |
| rs4149573 | 12 | 6449384 | TNFRSF1A | 0.09 | 0.09 | 0.88 | 1.05 (0.58-1.91) |
| rs767455 | 12 | 6450945 | TNFRSF1A | 0.42 | 0.42 | 0.94 | 1.01 (0.71-1.44) |
| rs4149570 | 12 | 6451590 | TNFRSF1A | 0.43 | 0.42 | 0.94 | 1.01 (0.7-1.46) |
| rs11064145 | 12 | 6455098 | TNFRSF1A | 0.45 | 0.43 | 0.79 | 1.05 (0.74-1.49) |
| rs2228576 | 12 | 6457062 | TNFRSF1A | 0.34 | 0.36 | 0.74 | 0.94 (0.64-1.37) |
| rs3764875 | 12 | 6458084 | TNFRSF1A | 0.42 | 0.35 | 0.15 | 1.31 (0.91-1.88) |
| rs11836136 | 12 | 110006512 | MVK | 0.33 | 0.26 | 0.06 | 1.43 (0.98-2.1) |
| rs7957619 | 12 | 110013879 | MVK | 0.16 | 0.11 | 0.08 | 1.54 (0.95-2.5) |
| rs11067376 | 12 | 110027795 | MVK | 0.4 | 0.36 | 0.38 | 1.18 (0.81-1.72) |
| rs2270374 | 12 | 110029186 | MVK | 0.15 | 0.18 | 0.26 | 0.76 (0.46-1.23) |
| rs17183465 | 12 | 110029750 | MVK | 0.15 | 0.18 | 0.37 | 0.8 (0.5-1.3) |
| rs10161126 | 12 | 110042348 | MVK | 0.44 | 0.47 | 0.45 | 0.88 (0.62-1.24) |
| rs1541597 | 12 | 110044918 | MVK | 0.24 | 0.2 | 0.29 | 1.25 (0.82-1.9) |
| rs5862 | 17 | 5402957 | NLRP1 | 0.46 | 0.47 | 0.79 | 0.95 (0.65-1.38) |
| rs8079034 | 17 | 5412361 | NLRP1 | 0.22 | 0.2 | 0.49 | 1.17 (0.75-1.83) |
| rs12602960 | 17 | 5414326 | NLRP1 | 0.29 | 0.26 | 0.52 | 1.13 (0.77-1.67) |
| rs4790267 | 17 | 5415287 | NLRP1 | 0.37 | 0.4 | 0.49 | 0.88 (0.62-1.26) |
| rs9911319 | 17 | 5415623 | NLRP1 | 0.38 | 0.45 | 0.19 | 0.77 (0.53-1.13) |
| rs9900356 | 17 | 5419278 | NLRP1 | 0.25 | 0.22 | 0.5 | 1.16 (0.75-1.78) |
| rs6502867 | 17 | 5420328 | NLRP1 | 0.24 | 0.24 | 0.91 | 1.03 (0.66-1.58) |
| rs12937224 | 17 | 5424042 | NLRP1 | 0.49 | 0.45 | 0.33 | 1.19 (0.84-1.68) |
| rs2301582 | 17 | 5436263 | NLRP1 | 0.4 | 0.39 | 0.77 | 1.06 (0.72-1.55) |
| rs11652907 | 17 | 5449665 | NLRP1 | 0.12 | 0.16 | 0.21 | 0.69 (0.39-1.24) |
| rs4790777 | 17 | 5451162 | NLRP1 | 0.39 | 0.39 | 0.87 | 1.03 (0.71-1.51) |
| rs12952888 | 17 | 5458824 | NLRP1 | 0.04 | 0.05 | 0.58 | 0.76 (0.27-2.07) |
| rs961826 | 17 | 5483937 | NLRP1 | 0.48 | 0.46 | 0.65 | 1.08 (0.76-1.54) |
| rs11078575 | 17 | 5485785 | NLRP1 | 0.45 | 0.46 | 0.82 | 0.96 (0.66-1.39) |
| rs871928 | 17 | 5486800 | NLRP1 | 0.15 | 0.17 | 0.53 | 0.85 (0.5-1.42) |
| rs925596 | 17 | 5488099 | NLRP1 | 0.13 | 0.11 | 0.43 | 1.25 (0.72-2.16) |

CHR=Chromosome, MAF=minor allele frequency, OR=Odds ratio, CI=confidence interval

Supplementary Table 5 Association analysis in JIA cases (not including systemic JIA cases) and controls for SNPs in *IL1 ligand* cluster and *IL1 receptor*

| SNP | CHR | Position | Gene | Major  allele | Minor  allele | MAF  Case | MAF  Control | p-value | Odds ratio  95% CI |
| --- | --- | --- | --- | --- | --- | --- | --- | --- | --- |
| rs2190360 | 2 | 102576098 | il1r | A | G | 0.35 | 0.35 | 0.94 | 1.0  (0.89-1.13) |
| rs6712572 | 2 | 113520678 | il1ligand | G | T | 0.51 | 0.5 | 0.62 | 1.04  (0.9-1.19) |
| rs2071374 | 2 | 113537352 | il1ligand | A | C | 0.29 | 0.27 | 0.12 | 1.1  (0.97-1.25) |
| rs1688075 | 2 | 113858196 | il1ligand | A | C | 0.08 | 0.07 | 0.37 | 1.14  (0.86-1.49) |

CHR=Chromosome, MAF=minor allele frequency, OR=Odds ratio, CI=confidence interval
